# Science Must Be Responsible to Society, Not to Politics

**DOI:** 10.1371/journal.pmed.1000222

**Published:** 2010-01-26

**Authors:** The PLoS Medicine Editors

## Abstract

The *PLoS Medicine* Editors discuss how collisions between science and politics slow progress in public health.

Governments rightly request the advice of scientists on matters of fact that affect the public good, from climate change to cancer screening. Scientists must then assess available data and present recommendations based on the data. But what is the role of scientists when politicians see these recommendations as inconvenient?

In mid-November 2009 the United States Preventive Services Task Force (USPSTF), which reviews scientific evidence to develop recommendations for the US health care community, revised their recommendations to say that women need not generally begin mammographic screening for breast cancer until age 50 [1]. This revision, which brings the Task Force's recommendations closer to those of the WHO, the UK, and the American College of Physicians, touched off a political firestorm in the US. The new recommendations amounted to a change in the evidence-based rating on screening women between ages 40 and 50 from grade B (recommended as likely to be of moderate benefit) to grade C (recommended against as a routine service, as net benefit is likely to be small, although there may be considerations that support providing the service in an individual patient). The US news media erupted in reports that this change in rating had confused women, ignored expert opinion, and perhaps even revealed government intentions to ration health care. Taken by surprise and facing potential embarrassment over an issue related to health care reform, the Obama administration distanced itself from the Task Force, with the Secretary of Health and Human Services commenting that its members had been appointed by the Bush administration. The Task Force members, some of whom took exception to this categorization, stood by their findings but admitted that they might have done a better job of communicating them.

The Task Force, composed of primary care clinicians with expertise in disease prevention and evidence-based medicine and convened under the federal Agency for Health Research and Quality (AHRQ), has been developing practical recommendations [2] since 1984. These recommendations can influence which preventive interventions US insurance plans will cover. Established to be independent of shifting political winds and private interests, the Task Force provides a model for how comparative effectiveness research to improve the evidence base for practicing medicine can proceed in a country where the influence of commercial interests on clinical guidelines is an ongoing issue.

A few days after the recommendations' release, Task Force Vice Chair Diana Petitti stated: “So, what does this mean if you are a woman in your 40s? You should talk to your doctor and make an informed decision about whether mammography is right for you based on your family history, general health, and personal values” [3]. Nonetheless, within weeks of the November guideline announcement, the US Senate acted unanimously to amend its health care bill to repudiate the Task Force recommendation [4]. One might argue that this action was necessary in the face of popular outcry to avoid irreversible damage to the greater goal of health care reform. Yet the very health care bill that the Senate amended already included language providing that comparative effectiveness research should proceed not along the existing model of unbiased panels within AHRQ, but under a new nongovernment institute overseen by a committee of 19 members including six from the private insurance and pharmaceutical industries and potentially only one from AHRQ itself [5]. It appears that the legislators, having found it politically advisable in the short term to second-guess the Task Force on the scientific evidence for breast cancer screening, were prepared in the long term to limit the role of unbiased experts in assessing medical evidence more broadly, apparently with an expectation that expanded input from the industries that profit most from health care would improve the process. Surely evidence-based medicine deserves better than a push out of the frying pan of partisan politics into the fire of vested interests.

The US government was not the only one to find itself in political turmoil over the medical evidence base. A few weeks prior to the USPSTF announcement, the UK Home Secretary asked David Nutt to resign as chair of the Advisory Council on the Misuse of Drugs [6]. Nutt, a professor at Imperial College London, had given a lecture challenging the classification scheme that determines legal penalties for drug use in the UK, noting that the classification of some drugs does not reflect the evidence for their potential to cause harm. (For example, harm ratings rank alcohol and tobacco as more harmful than the illegal drugs LSD, ecstasy, and cannabis [7].) Nutt subsequently commented, “I have repeatedly stated [cannabis] is not safe, but that the idea that you can reduce use through raising the classification in the Misuse of Drugs Act from class C to class B—where it had previously been placed, but thus now increasing the maximum penalty for possession for personal use to 5 years in prison—is implausible” [8]. The Home Office, for their part, expressed “surprise and disappointment over Professor Nutt's comments which damage efforts to give the public clear messages about the dangers of drugs,” and confirmed that “we remain determined to crack down on all illegal substances and minimise their harm to health and society as a whole.” Here, as in the case of cancer screening in the US, the conflict appears to be less about the quality of the evidence than about how – and even whether – scientists are to communicate evidence on politically volatile topics to the public.

In an enlightened society, surely science must serve and enhance the public good, and even the best medical research will not attain this goal until its significance reaches policymakers and the public in a way that leads to improvements in health. If science is to offer anything over opinion polls, scientists must report unbiased observations in an objective fashion, whether or not the data are comforting, expected, or even easily understood. Scientists cannot selectively emphasize the aspects of their work that will meet with the widest approval, as politicians sometimes do. Nonetheless, scientists—particularly those invited by governments to provide expertise—bear responsibility for communicating their work with sensitivity to its context and anticipated impact. This kind of attention to public interpretation of their work may not be what many scientists are trained for or desire, but without it there seems little hope that scientific evidence—particularly when it conflicts with the goals of politicians—will emerge beyond barriers of indifference, suspicion, or even hostility and appropriately inform policy.

But however diligently scientists work to ensure the integrity of their work and the accuracy of its public perception, scientists alone cannot realize the potential of science to improve society. For real progress to occur, those with power to implement change must act on the evidence. Politicians must not ignore nor attempt to discredit legitimate science that doesn't happen to support their political goals; to do so erodes public trust not only in scientists but in politicians themselves. In the case of health research, politicians must remember that society encompasses not only the corporate engines of economic growth and decline, but also individuals whose lives depend on the quality of health care data.

If governments are to engage in reform that improves health, and not only the economic structures through which health care is provided, those governments must develop and support systems to judge the quality of health research independent of vested interests and political expedience. Informed citizens should expect and require their governments to continue inviting independent scientists on board, to heed their advice in navigating, and not to jettison scientific evidence when weathering a political storm.

## Abbreviations

AHRQ: Agency for Health Research and Quality
USPSTF: United States Preventive Services Task Force
